# Supplementary material for: Modeling glioblastoma heterogeneity as a dynamic network of cell states
Source: Mol Syst Biol. 2021 Sep 16;17(9):e10105. doi: 10.15252/msb.202010105 (PMC8444284; doi:10.15252/msb.202010105)
Supplement: Supplementary file 6 — Source Data for Figure 5 [file MSB-17-e10105-s004.zip › Figure5A_sourcedata/GSEA_3017/nosigdb_stateA.GseaPreranked.1622795026485/RICHARDS_ZHONG_NATURE_2018_NPCS_UPREG.html]

Details for gene set RICHARDS\_ZHONG\_NATURE\_2018\_NPCS\_UPREG[GSEA]

|  || Dataset | state53017 |
| Phenotype | NoPhenotypeAvailable |
| Upregulated in class | na\_pos |
| GeneSet | RICHARDS\_ZHONG\_NATURE\_2018\_NPCS\_UPREG |
| Enrichment Score (ES) | 0.4765477 |
| Normalized Enrichment Score (NES) | 3.2747202 |
| Nominal p-value | 0.0 |
| FDR q-value | 0.0 |
| FWER p-Value | 0.0 |
Table: GSEA Results Summary

  

Fig 1: Enrichment plot: RICHARDS\_ZHONG\_NATURE\_2018\_NPCS\_UPREG      
 Profile of the Running ES Score & Positions of GeneSet Members on the Rank Ordered List

  

| PROBE | GENE SYMBOL | GENE\_TITLE | RANK IN GENE LIST | RANK METRIC SCORE | RUNNING ES | CORE ENRICHMENT || 1 | CDK1 |  |  | 3 | 1.006 | 0.0289 | Yes |
| 2 | MKI67 |  |  | 4 | 0.926 | 0.0585 | Yes |
| 3 | KIF11 |  |  | 6 | 0.870 | 0.0852 | Yes |
| 4 | UBE2C |  |  | 7 | 0.793 | 0.1105 | Yes |
| 5 | BUB3 |  |  | 12 | 0.740 | 0.1298 | Yes |
| 6 | KPNA2 |  |  | 20 | 0.667 | 0.1435 | Yes |
| 7 | HIST1H4C |  |  | 21 | 0.659 | 0.1645 | Yes |
| 8 | ASPM |  |  | 27 | 0.639 | 0.1795 | Yes |
| 9 | CCNB1 |  |  | 37 | 0.599 | 0.1888 | Yes |
| 10 | CKS2 |  |  | 40 | 0.587 | 0.2053 | Yes |
| 11 | VIM |  |  | 46 | 0.543 | 0.2172 | Yes |
| 12 | HJURP |  |  | 47 | 0.539 | 0.2345 | Yes |
| 13 | CKS1B |  |  | 50 | 0.530 | 0.2492 | Yes |
| 14 | MIS18BP1 |  |  | 64 | 0.495 | 0.2508 | Yes |
| 15 | CCNA2 |  |  | 65 | 0.495 | 0.2666 | Yes |
| 16 | TPX2 |  |  | 66 | 0.488 | 0.2822 | Yes |
| 17 | DLGAP5 |  |  | 76 | 0.475 | 0.2876 | Yes |
| 18 | CDC20 |  |  | 80 | 0.472 | 0.2994 | Yes |
| 19 | UBE2T |  |  | 83 | 0.470 | 0.3122 | Yes |
| 20 | DUT |  |  | 87 | 0.466 | 0.3238 | Yes |
| 21 | TUBB4B |  |  | 93 | 0.462 | 0.3331 | Yes |
| 22 | CENPF |  |  | 94 | 0.461 | 0.3479 | Yes |
| 23 | TOP2A |  |  | 95 | 0.457 | 0.3625 | Yes |
| 24 | ZWINT |  |  | 106 | 0.447 | 0.3658 | Yes |
| 25 | TGIF1 |  |  | 107 | 0.447 | 0.3801 | Yes |
| 26 | AURKB |  |  | 108 | 0.446 | 0.3943 | Yes |
| 27 | KIF23 |  |  | 116 | 0.436 | 0.4006 | Yes |
| 28 | MELK |  |  | 123 | 0.427 | 0.4077 | Yes |
| 29 | HELLS |  |  | 135 | 0.417 | 0.4090 | Yes |
| 30 | GTSE1 |  |  | 146 | 0.411 | 0.4112 | Yes |
| 31 | CCNB2 |  |  | 147 | 0.411 | 0.4244 | Yes |
| 32 | MAD2L1 |  |  | 153 | 0.406 | 0.4319 | Yes |
| 33 | KIF2C |  |  | 165 | 0.397 | 0.4326 | Yes |
| 34 | MCM7 |  |  | 171 | 0.394 | 0.4397 | Yes |
| 35 | NDC80 |  |  | 173 | 0.393 | 0.4512 | Yes |
| 36 | BUB1 |  |  | 188 | 0.384 | 0.4482 | Yes |
| 37 | KIF22 |  |  | 197 | 0.377 | 0.4515 | Yes |
| 38 | CDCA3 |  |  | 203 | 0.372 | 0.4579 | Yes |
| 39 | SMC4 |  |  | 217 | 0.367 | 0.4554 | Yes |
| 40 | SPC25 |  |  | 226 | 0.363 | 0.4583 | Yes |
| 41 | H2AFZ |  |  | 245 | 0.351 | 0.4498 | Yes |
| 42 | FEN1 |  |  | 259 | 0.343 | 0.4466 | Yes |
| 43 | KNSTRN |  |  | 263 | 0.340 | 0.4542 | Yes |
| 44 | CLSPN |  |  | 274 | 0.336 | 0.4540 | Yes |
| 45 | DEPDC1B |  |  | 275 | 0.335 | 0.4647 | Yes |
| 46 | ORC6 |  |  | 283 | 0.333 | 0.4677 | Yes |
| 47 | C21orf58 |  |  | 294 | 0.328 | 0.4672 | Yes |
| 48 | CENPN |  |  | 310 | 0.323 | 0.4611 | Yes |
| 49 | HSPB1 |  |  | 312 | 0.322 | 0.4703 | Yes |
| 50 | SFRP2 |  |  | 320 | 0.319 | 0.4729 | Yes |
| 51 | CDCA5 |  |  | 341 | 0.312 | 0.4610 | Yes |
| 52 | CLIC1 |  |  | 342 | 0.311 | 0.4709 | Yes |
| 53 | PRC1 |  |  | 350 | 0.310 | 0.4732 | Yes |
| 54 | CKAP2 |  |  | 379 | 0.301 | 0.4522 | Yes |
| 55 | RPA3 |  |  | 381 | 0.300 | 0.4607 | Yes |
| 56 | RRM2 |  |  | 383 | 0.300 | 0.4692 | Yes |
| 57 | CENPW |  |  | 386 | 0.299 | 0.4765 | Yes |
| 58 | CREB5 |  |  | 407 | 0.291 | 0.4640 | No |
| 59 | HMGB2 |  |  | 424 | 0.288 | 0.4557 | No |
| 60 | NUSAP1 |  |  | 430 | 0.286 | 0.4594 | No |
| 61 | NUF2 |  |  | 434 | 0.285 | 0.4652 | No |
| 62 | ANP32E |  |  | 453 | 0.280 | 0.4545 | No |
| 63 | ATAD2 |  |  | 470 | 0.275 | 0.4458 | No |
| 64 | KIF15 |  |  | 538 | 0.263 | 0.3810 | No |
| 65 | CDKN3 |  |  | 539 | 0.263 | 0.3894 | No |
| 66 | FBXO5 |  |  | 557 | 0.258 | 0.3790 | No |
| 67 | SOX9 |  |  | 577 | 0.254 | 0.3664 | No |
| 68 | TMPO |  |  | 584 | 0.253 | 0.3679 | No |
| 69 | NOTCH1 |  |  | 666 | -0.280 | 0.2883 | No |
| 70 | RPS27L |  |  | 688 | -0.295 | 0.2748 | No |
| 71 | ZFHX4 |  |  | 700 | -0.301 | 0.2724 | No |
| 72 | HES1 |  |  | 736 | -0.331 | 0.2448 | No |
| 73 | TAGLN2 |  |  | 963 | -0.891 | 0.0262 | No |
Table: GSEA details [plain text format]

  

Fig 2: RICHARDS\_ZHONG\_NATURE\_2018\_NPCS\_UPREG: Random ES distribution      
 Gene set null distribution of ES for **RICHARDS\_ZHONG\_NATURE\_2018\_NPCS\_UPREG**
